# *S*-Adenosylmethionine (SAMe) in major depressive disorder (MDD): a clinician-oriented systematic review

**DOI:** 10.1186/s12991-020-00298-z

**Published:** 2020-09-05

**Authors:** Alessandro Cuomo, Bruno Beccarini Crescenzi, Simone Bolognesi, Arianna Goracci, Despoina Koukouna, Rodolfo Rossi, Andrea Fagiolini

**Affiliations:** 1 Division of Psychiatry, Department of Molecular Medicine, University of Siena, Siena, Italy; 2 Department of Systems Medicine, University of Rome tor Vergata, Rome, Italy

**Keywords:** Systematic review, *S*-Adenosylmethionine, Antidepressant, Depression, Nutraceutical

## Abstract

**Background:**

Major depressive disorder (MDD) is a recurrent illness with high rates of chronicity, treatment-resistance, and significant economic impact. *S*-Adenosylmethionine (SAMe), a molecule that is formed naturally in the human body, has shown antidepressant effects and may expand the available options for treating MDD. This systematic review examines the evidence concerning the efficacy of SAMe as monotherapy or in combination with antidepressants.

**Methods:**

A systematic search in Medline, Psychinfo, AMED, and Cochrane Controlled Trials Register was conducted for any reference recorded up to March 2020. Double-blind, randomised controlled trials, comparing the antidepressant efficacy of SAMe to placebo or/and to other antidepressants, were selected. Two authors evaluated each study independently and then, reconciled findings.

**Results:**

Eight trials, with a total of 11 arms and 1011 subjects, evaluating the efficacy of SAMe used as monotherapy or as adjunctive therapy (512 individuals), were included in this review. The study duration ranged between 2 and 12 weeks and the daily dose of SAMe varied from 200 to 3200 mg. Five comparisons evaluated the differences between SAMe and placebo and SAMe resulted significantly better than placebo in three of these studies. Four comparisons evaluated the differences between SAMe and other antidepressants (imipramine or escitalopram) and showed no significant difference. One study showed that SAMe was significantly better than placebo in accelerating the response to imipramine from day 4 to day 12, but the mean scores were not statistically different at the day 14 endpoint. One study showed that SAMe combined with serotonin reuptake inhibitors (SSRI) was better than PBO combined with SSRI. The studies reported only mild, transient or non-clinically relevant side effects.

**Conclusions:**

The existing trials of SAMe, used as monotherapy or add on to another antidepressants, have shown encouraging and generally positive results. However, more evidence is necessary before definitive conclusions can be drawn. Larger, double-blind randomised controlled studies are warranted to confirm the antidepressant effectiveness of SAMe.

## Background

Major depressive disorder (MDD) is the fourth leading cause of global disease burden [1]. Approximately 40–50% of patients do not achieve an adequate response after initial treatment [2, 3], and full remission is too often short-lived or absent [4]. Nutraceutical (e.g. omega-3, *S*-adenosylmethionine, or vitamin D), used in augmentation or combination with antidepressants, may represent an effective and safe strategy in enhancing antidepressants effects [5–9].

*S*-Adenosylmethionine (SAMe), an endogenous compound that is not readily available from dietary sources [9, 10], was discovered in 1952 by the late Italian scientist, Giulio Cantoni [10], marketed since 1999 as a dietary supplement and, then, as an antidepressant [11].

SAMe is a natural sulphur-containing compound with a reactive methyl group [11]. The synthesis of SAMe includes the methylation of homocysteine to methionine by methyltransferase enzyme and cobalamin. Methionine is then converted to SAMe through the enzyme methionine adenosyltransferase. SAMe may play a beneficial role in biochemical mechanisms that have been associated with depression. For instance, SAMe may affect the regulation of a wide range of critical components of neurotransmission [11–17]. SAMe is involved in three central metabolic pathways, namely trans-sulfuration (synthesis of glutathione), transaminopropylation (development of polyamines), and methylation (synthesis of sarcosine; conversion of norepinephrine to epinephrine; catabolism and anabolism of monoaminergic neurotransmitters [11, 12, 16, 17]. Several studies have observed the dysregulation of the one-carbon metabolism, and lower levels of methionine adenosyltransferase enzyme, cerebrospinal fluid SAMe and methylation deficit in patients with MDD [11–14]. Worthy of consideration is also the possibility that SAMe enhances gene expression of brain-derived neurotrophic factor [11, 18].

SAMe has proved effective in several studies involving patients with MDD [10, 11, 21–28]. As for other nutraceuticals, SAMe’s optimal dose for depression is still unknown, but oral doses tend to be on average 1600 mg/day, while parental doses range between 200 and 400 mg/day [20, 29]. To date, oral SAMe dose–response relationship is still unknown although it has been suggested that patients failing to respond to 1600 mg/day may improve after increasing their dose to 3200 mg/day [30]. Tolerability is usually good, but an increased risk of manic–hypomanic switch has been reported, especially with the parental formulation and for patients with a diagnosis of bipolar disorder [21, 31–35].

A number of studies have investigated the efficacy of SAMe for the treatment of depression and the aim of this paper is to provide an updated overview of the available evidence about the efficacy and tolerability of SAMe, as monotherapy or adjunctive treatment for the treatment of MDD. Our goal is to offer a summary to clinicians interested in prescribing these medications and to identify research gaps or open clinical questions, which may stimulate more studies. Our hypothesis was that the overall results of existing trials were substantially positive, but more research and evidence is needed before the antidepressant effectiveness of SAMe is definitively supported.

## Methods

**Fig. 1:**
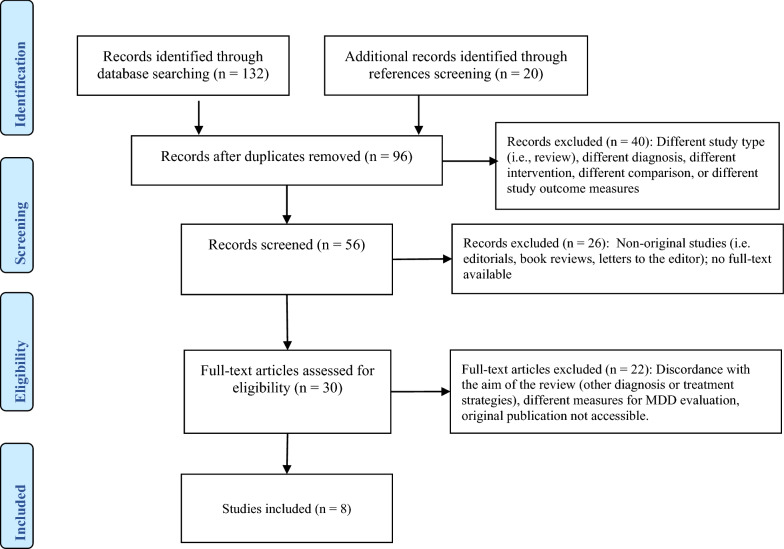
Prisma flowchart for article selection

A systematic search on Medline, Psychinfo, AMED, and Cochrane Controlled Trials Register was conducted on all references published up to March 2020. The search keywords were: SAMe (or *S*-adenosyl methionine or *s*-adenosyl-l-methionine) and major depressive disorder, MDD, depression, and perinatal depression. We included RCTs and considered published and unpublished trials comparing the effectiveness of SAMe versus placebo or active agents, or SAMe combined with other antidepressants in the treatment of MDD. No language or time restrictions were applied. Two authors (AF, AC) screened the results of the search using an over-inclusive approach to construct a list of all potentially relevant articles. The same authors independently screened abstracts, examined the full-text of the most relevant papers. We did not calculate a kappa statistic for measuring the agreement between the two authors because there was not any disagreement. One hundred thirty-two articles were identified, and the cross-check of their references revealed 20 more articles. Fifty-six duplicates were removed from the resulting 152 manuscripts, 40 studies were excluded after reading the abstract (different diagnosis, comparison, intervention, or outcome); subsequently, 26 studies were excluded because they were not original studies or full-text was not available. Finally, 22 more papers were excluded after full-text revision because they included other diagnoses or treatment strategies. Eight double-blind clinical studies were included in this review (Prisma flowchart—Fig. 1).

## Data extraction

Two authors (AF, AC) independently extracted relevant data from the selected studies. Extracted data were related to one of the following comparisons: SAMe versus placebo as monotherapy, SAMe versus another antidepressant as monotherapy, and SAMe versus placebo as adjunctive treatments to another antidepressant (imipramine or serotonin reuptake inhibitors) (Table 1).

**Table 1:** Characteristics of the included RCTs of SAMe in MDD

Author	Study typology	Duration	Dose SAMe	Administration	Primary outcome	Diagnostic criteria
Caruso 1984 [36]	SAMe vs PBO	3 weeks	200 mg/day	IM	HAM-D (21 items)	Not specified
Kagan 1990 [37]	SAMe vs PBO	3 weeks	200 mg/day	OR	HAM-D (21 items)	DSM-III
Berlanga 1992 [38]	SAMe + IMI vs PBO + IMI	2 weeks	200 mg/day	IM	HAM-D (17 items)	DSM-III-R
Papakostas 2010 [39]	SAMe + SRI vs PBO + SRI	6 weeks	800 mg/day	OR	HAM-D	DSM-IV
Delle Chiaie 2002a [40]	SAMe vs IMI	6 weeks	1600 mg/day	OR	HAM-D (21 items)	DSM-IV
Delle Chiaie 2002b [40]	SAMe vs IMI	4 weeks	400 mg/day	IM	HAM-D (21 items)	DSM-IV
Mischoulon 2014a [41]	SAMe vs PBO	12 weeks	1600–3200 mg/day	OR	HAM-D (17 items)	IDS-C ≥ 25
Mischoulon 2014b [41]	SAMe vs EST	12 weeks	1600–3200 mg/day	OR	HAM-D (17 items)	IDS-C ≥ 25
Sarris 2014a [42]	SAMe vs PBO	12 weeks	1600 mg/day	OR	HAM-D (17 items)	DSM-IV
Sarris 2014b [42]	SAMe vs EST	12 weeks	1600 mg/day	OR	HAM-D (17 items)	DSM-IV
Sarris 2018 [43]	SAMe vs PBO	8 weeks	800 mg/day	OR	MADRS	DSM-V

*IM* intramuscular, *OR* oral, *PBO* placebo, *IMI* imipramine, *EST* escitalopram, *MDD* major depressive disorder, *SAMe* *S*-Adenosylmethionine, *SRI* serotonin reuptake inhibitors

## Results

Eight double-blind randomised and controlled studies were examined. The trials were conducted from 1984 to 2018 and all subjects had a diagnosis of MDD. The number of comparisons was 11, given that three of the eight trials [40–42] included three arms. Two of these trials compared SAMe, escitalopram and placebo [41, 42]. The remaining study [43] included two multicenter trials, respectively, comparing SAMe oral (first multicenter trial) and SAMe intramuscular (second multicenter trial) to imipramine.

Five comparisons evaluated the differences between SAMe and placebo [36, 37, 41–43]; four comparisons evaluated the differences between SAMe and another antidepressant (imipramine or escitalopram) [40–42]; two comparisons evaluated the differences between SAMe and placebo, as adjunctive treatments on top of another antidepressant [38, 39].

In 10 of the 11 comparisons, treatment efficacy was determined by the Hamilton Depression Rating Scale (HAM-D). The remaining trial [43] used the Montgomery–Asberg Depression Rating Scale (MADRS) as the primary outcome measure (Table 1). Response to treatment was defined as a reduction of 50% or more of HAM-D or MADRS.

The mean study duration was 7.3 ± 4.1 weeks and the SAMe dose ranged between 200 to 3200 mg/day. Most studies used oral SAMe formulation; three studies tested SAMe intramuscular formulation [36, 38, 40].

The total number of subjects included in the trials was 1011. Five hundred twelve patients received SAMe alone (468 patients) or combined with imipramine or SRI (44 patients). One hundred sixteen patients received placebo, 20 patients received placebo and imipramine, 31 received placebo and SRI, and 332 received either imipramine or escitalopram (282 imipramine intramuscularly or by mouth, and 50 escitalopram by mouth).

## Efficacy

Table 2 reports a summary of the efficacy and tolerability data in the trials that are reported in this paper.

**Table 2:** Summary of efficacy and tolerability data

Study	SAMe sample	Control sample	MD SAMe	MD control	Efficacy	Side effects	Response method	Response rate SAMe	Response rate control
Caruso 1984 [36]	25	24	11.4 (7.7)	2.9 (6.4)	SAMe better than placebo	Not reported	NR	NR	NR
Kagan 1990 [37]	9	6	16.2 (6.9)	7.3 (14.3)	SAMe better than placebo	Mild side effects only, resolved spontaneously	NR	NR	NR
Berlanga 1992 [38]	20	20	13.7 (4.6)	9.6 (6.2)	SAMe + IMI better than placebo + IMI	Typical anticholinergic effects of IMI (mild). Mild weight gain	Reduction HAM ≥ 50%	65	45
Papakostas 2010 [39] *	24	31	11.1 (6.1)	15.8 (6.2)	SAMe + SRI better than placebo + SRI	Slightly higher mean supine systolic blood pressure in the treatment group	Reduction HAM ≥ 50%	75.0	12.9
Delle Chiaie 2002a [40]	143	135	12.6 (8.1)	13.1 (8.6)	SAMe equal to imipramine	Dry mouth, constipation, and tachycardia. More frequent IMI	Reduction HAM ≥ 50%	51	57
Delle Chiaie 2002b [40]	146	147	12.6 (8.0)	13.1 (7.1)	SAMe equal to imipramine		Reduction HAM ≥ 50%	58.9	50.3
Mischoulon 2014a [41]	36	31	6.19 (7.38)	5.11 (6.92)	SAMe equal to placebo	Good tolerability. GI complaints	Reduction HAM ≥ 50%	35	30
Mischoulon 2014b [41]	36	30	6.19 (7.38)	6.31 (6.98)	SAMe equal to escitalopram		Reduction HAM ≥ 50%	35	34
Sarris 2014a [42]	18	16	7.31 (5.96)	4.0 (5.6)	SAMe better than placebo	Interventions were well tolerated with no significant adverse effects	Reduction HAM ≥ 50%	45	26
Sarris 2014b [42]	18	20	7.31 (5.96)	6.69 (5.7)	SAMe equal to escitalopram		Reduction HAM ≥ 50%	45	31
Sarris 2018 [43]	37	39	11.4 (7.54)	12.1 (7.02)	SAMe equal to placebo	Irritability, hot/cold flashes, cramping, confusion, headache, vertigo/dizziness, twitching, sleep difficulty and fatigue	Reduction MADRS ≥ 50%	54.3	50

*NR* not reported, *MD* mean difference

### Comparison 1: SAMe versus placebo as monotherapy

Five studies compared SAMe and placebo, for a total of 125 patients treated with SAMe as monotherapy and 116 patients treated with placebo. HAM-D score decreased significantly in three out of the five studies [36, 37, 42]. Caruso et al. [36] reported a significant improvement of depression using SAMe at 200 mg/day in intramuscular formulation (SAMe mean difference: 11.4; placebo mean difference: 2.9); Kagan et al. [37] and Sarris et al. [42] reported a significant improvement using SAMe oral formulation at 800 mg/day and 1600 mg/day, respectively.

In a 12-week study [41], patients were randomly assigned to SAMe 1600 mg/day or placebo for the first 6 weeks. From the sixth week, the doses were escalated to maximise the probability of response in the event of no-response. The authors observed a statistically significant decrease in HAM-D score for the SAMe group until the tenth week of treatment (*p* = 0.026). However, the statistical significance was lost at the study end point (12th week) [41]. Finally, Sarris et al. [43] reported that SAMe was not significantly better than placebo, in a study where SAMe was administered orally at 800 mg/day.

### Comparison 2: SAMe versus imipramine and SAMe versus escitalopram as monotherapy

Four studies compared SAMe monotherapy efficacy with imipramine or escitalopram, with a total number of 343 patients that received SAMe and 332 treated with the other antidepressants. No statistically significant difference emerged [40–42].

### Comparison 3: SAMe versus placebo as adjunctive therapy

Two studies [38, 39] analysed SAMe efficacy as adjunctive treatment antidepressant. The trials included a total of 95 subjects (SAMe: 44; placebo: 51). In one of the studies, SAMe resulted significantly better than placebo in accelerating the response to imipramine from fourth to the twelfth day, while the mean HRSD scores no longer differed significantly at day 14 [38]. In the other study, SAMe combined to SSRI was better than SSRI alone in patients with MDD not-responding to SSRIs [39].

### Safety and tolerability

SAMe tolerability was good for most studies. Side effects were only mild and transient. Two studies [39, 43] reported, respectively, two and three withdrawals in the placebo arm, while five and two patients withdrew because of adverse events in the SAMe arm. Typical anticholinergic effects of imipramine (dry mouth, constipation, and tachycardia) and other relatively mild side effects were registered, respectively, in patients treated with IMI [38, 40] or SSRI [39], in monotherapy or in combination with SAMe. For the oral formulation, common mild side effects are gastrointestinal symptoms, sweating, vertigo, dizziness, tachycardia, restlessness, and anxiety [5, 11, 20]. Patients treated with doses higher than 1600 mg/day reported stomach and abdominal discomfort, fluid retention and swelling [30].

## Discussion

Many patients affected by MDD continue to be symptomatic despite second, third, or fourth-line treatment approaches [44] and SAMe may represent a useful aid for the treatment for MDD, especially in those cases where the risk–benefit ratio may not justify the use of less-tolerated pharmacological treatment [5, 10]. SAMe’s mechanism of action is still unclear, but it has been shown that SAMe is able to increase the central turnover rate of dopamine and serotonin [38]. In fact, SAMe raises cerebrospinal fluid levels of both homovanillic acid and 5-hydroxyindoleacetic acid, while lowering the levels of serum prolactin [36]. SAMe is able to impact on the noradrenergic system as well. An increase in the number of beta-adrenergic receptors and in the affinity of alpha1-adrenergic receptors for the agonist phenylephrine has been observed in rats, after the administration of SAMe [45]. Hence, the administration of SAMe leads to modifications in adrenergic neurotransmission that are opposite to those that are classically produced by standard antidepressants: upward regulation of alpha-adrenergic receptors and downward regulation of beta-adrenergic receptors. Of interest, antidepressant treatments may lead to a depletion of SAMe’s concentration in tissues [45], which may be replaced by the administration of more SAMe. Indeed, SAMe’s mechanism of action likely involves different neurochemical effects, including enhanced methylation of catecholamines and increased serotonin turnover, reuptake inhibition of norepinephrine, enhanced dopaminergic activity, decreased prolactin secretion, and increased phosphatidylcholine conversion [19, 46].

The role of *S*-adenosylmethionine in the treatment of major depressive disorder has been established in clinical studies [5, 20, 35], but some uncertainties remain [34]. Overall, SAMe has demonstrated the ability to induce a valid and effective antidepressants effect, with remarkably few side effects and a relatively rapid onset of action [24]. However, the number of trials that have been conducted to date is relatively small and more trials, involving a greater number of subjects, are needed to definitively establish SAMe effectiveness.

To date, other nutraceuticals, like folic acid, methyl folate, omega-3, and vitamin D, are available and could represent an alternative to SAMe use. However, they are not without risk [47]. For instance, supplements containing folic acid or omega-3 could increase risk of prostate cancer and higher-dose omega-3 supplementation has been suspected to increase bleeding, impair immune function and metabolism [48–50]. Vitamin D could determine hypercalcemia and vascular calcification if used at high doses (≥ 275 mg/day) [35].

## Limitations

Our review has several limitations due to the limited number of studies that have been published and analysed. Furthermore, the SAMe doses that were used in the included trials as well as the observation periods, inclusion criteria, and statistical methods, were not uniform across the studies, making it difficult compare the studies and lump the results together.

## Advantages of the revision

*S*-Adenosylmethionine (SAMe) represents a frequently used antidepressants agent and this revision provides clinicians with a summary of the existing findings and may stimulate researchers to conduct larger trials.

## Conclusions

SAMe, used as monotherapy or combined with other antidepressants, is well tolerated and may improve MDD symptoms. The existing findings are encouraging, but further, well-designed, randomised, controlled clinical trials are needed to provide definitive evidence about SAMe efficacy and tolerability, both as monotherapy and as adjunctive treatment.
